# Examining the impact of growth mindset and lifelong learning on teacher performance among university faculty: The mediating role of professional development

**DOI:** 10.1371/journal.pone.0341741

**Published:** 2026-02-05

**Authors:** Yuqiao Luo, Yu Bi, Lingjie Wang

**Affiliations:** 1 Department of Education Management, Chinese International College, Dhuraki Pundit University, Bangkok, Thailand; 2 Basic Course Department, Hengshui University, Hengshui, China; Universiti Pertahanan Nasional Malaysia, MALAYSIA

## Abstract

Based on social constructivist learning theory, this study constructs a structural model with teacher growth mindset and lifelong learning as independent variables, teacher professional development as a mediating variable, and teacher performance as the dependent variable, aiming to explore how cognitive beliefs and behavioral tendencies jointly influence teacher performance. Through a questionnaire survey of 620 university teachers and empirical testing using structural equation modeling (SEM), results show that growth mindset and lifelong learning are positively associated with teacher performance. Moreover, teacher professional development functions as an associational mediator linking both growth mindset and lifelong learning with performance. Given the cross-sectional design, these findings reflect statistical associations rather than causal effects. The research findings expand the theoretical model of factors influencing teacher performance and provide practical references for optimizing teacher professional development pathways and university human resource management.

## 1. Introduction

China’s higher education sector has undergone continuous transformation in recent years, driven by system-wide reforms and the implementation of the “Double First-Class” initiative. As universities pursue higher standards of teaching and research, faculty members are experiencing increasing expectations regarding instructional quality, scholarly productivity, and sustained professional growth [1]. These shifts have made the question of how to foster teachers’ ongoing development and support their performance a central concern for both institutional leaders and educational researchers [2,3]. Because teacher professional development is closely linked to the quality of teaching and learning, strengthening teachers’ professional growth is essential for enhancing overall performance in university classrooms [4,5].

A growing body of research has highlighted the importance of teachers’ psychological and behavioral characteristics in shaping their professional development [6,7]. Among these characteristics, growth mindset and lifelong learning readiness have attracted considerable scholarly attention [8,9]. Growth mindset, introduced by Dweck [8], reflects individuals’ beliefs that their abilities can be improved through effort and learning. Teachers with stronger growth mindsets tend to remain resilient when encountering instructional challenges, seek constructive feedback, and actively refine their teaching practices [10,11]. Lifelong learning, meanwhile, is widely recognized as a core professional competence for 21st-century teachers. It supports educators in adapting to technological innovation, updating pedagogical beliefs, and sustaining their professional vitality in rapidly changing teaching environments [12,13].

Social constructivist learning theory further provides a useful lens for understanding these phenomena. The theory posits that knowledge and capacity development are shaped through social interaction, cultural participation, and reflective engagement [14]. Within this framework, both growth mindset and lifelong learning orientation function as personal dispositions that become meaningful when enacted through teachers’ involvement in professional communities, instructional practice, and continuous reflection.

Although empirical studies have documented the relevance of growth mindset and lifelong learning for teacher development, most prior work has focused on compulsory education rather than higher education contexts [15]. Research in higher education has tended to emphasize the outcomes of professional development, while comparatively fewer studies have investigated the cognitive and motivational antecedents that shape university teachers’ professional learning engagement and performance [16]. This gap indicates the need to examine how university teachers’ psychological and behavioral characteristics are statistically associated with their performance, particularly through the pathway of professional development, which aligns with broader concerns about improving higher education quality.

To address this need, the present study proposes a structural equation model to examine the associations among teacher growth mindset, lifelong learning, professional development, and teacher performance. Using survey data and structural modeling techniques, this study aims to: (1) contribute empirical evidence to the literature on university teacher professional development by identifying the associational pathways linking cognitive and behavioral factors with performance; and (2) offer data-informed insights for universities seeking to improve faculty development and management practices.

## 2. Theoretical foundation and research hypotheses

### 2.1 Social constructivist learning theory

This study is based on Social Constructivist Learning Theory, which posits that knowledge acquisition stems from individual interaction and collaboration within sociocultural contexts [17]. Teachers are not isolated learners but continuously construct their professional capabilities through reflection, communication, and practice within specific teaching practices and professional communities. Growth mindset, as a belief emphasizing “developable abilities,” makes teachers more willing to accept challenges and participate in improvement, achieving self-growth through interaction with environments and peers. Lifelong learning represents teachers’ continuous participation in school-based research, professional training, and other activities, constituting a process of continuously constructing knowledge and updating concepts within social contexts. Meanwhile, teacher professional development, as a key associational pathway, also aligns with constructivism’s advocacy of “learning through practice. Teachers continuously optimize teaching behaviors through community collaboration, situational participation, and experiential reflection, thereby improving performance. Thus, growth mindset and lifelong learning show indirect associations with teacher performance through their links with professional development within sociocultural interaction contexts.

### 2.2 Teacher growth mindset, professional development, and teacher performance

#### 2.2.1 Teacher growth mindset and teacher performance.

Growth Mindset, proposed by Yeager and Dweck [18], refers to a cognitive tendency where individuals believe their abilities can be continuously developed through acquired effort [18]. Seaton [19] found through a questionnaire survey of 300 secondary school teachers that growth mindset was significantly associated with teachers’ teaching persistence and classroom goal-oriented behaviors [19]. Found in a structural equation model analysis of Chinese university teachers that growth mindset was not only positively associated with teachers’ teaching innovation behaviors, but also showed an indirect association with teaching performance through its relationship with professional learning motivation [20]. demonstrated through an empirical study of 243 university teachers in Taiwan that growth mindset has significant positive relationships with teachers’ participation in curriculum reform, teaching reflection, and student satisfaction [21]. Furthermore, Fathi et al. [22] indicated based on research with Iranian teacher groups that growth beliefs significantly correlate with teacher self-efficacy, which is considered an important psychological factor that is statistically associated with teacher performance. [22]. Recent international studies have examined teacher growth mindset across diverse educational contexts. For instance, Griful-Freixenet et al. [23] demonstrated that growth-oriented beliefs are associated with adaptability and engagement in learning-related behaviors. In the field of teacher education, Rissanen and Kuusisto [24] and Bardach et al. [25] further reported that teachers’ growth mindset is positively associated with professional learning engagement and openness to feedback, suggesting its relevance for ongoing professional development in different cultural settings.In summary, growth mindset is not only a psychological trait but also a key factor that can be transformed into teaching behaviors. Therefore, this study suggests that teacher growth mindset is significantly and positively associated with their performance.

#### 2.2.2 Teacher growth mindset and teacher professional development.

Teachers with growth mindset tend to view teaching failures as growth opportunities, thereby stimulating motivation for continuous improvement and autonomous learning [26], actively participating in school-based research, professional training, and reflective practice activities [27]. Growth mindset prompts teachers to firmly believe that abilities can be improved, making them more willing to accept new concepts, try new methods, and continuously optimize teaching behaviors, promoting professional capability development [28]. Additionally, growth mindset enhances teachers’ resilience and learning motivation, serving as important psychological support for professional growth [29], and is closely related to key traits such as reflective ability and self-efficacy [30]. From an international perspective, lifelong learning has been widely regarded as a core dimension of teacher professionalism. Empirical studies across different countries indicate that teachers’ lifelong learning orientation is associated with sustained engagement in professional development, reflective practice, and participation in learning communities [31,32]. International policy reports have also emphasized lifelong learning as a key component of teachers’ professional growth in contemporary education systems [33]. Therefore, this study suggests that teacher growth mindset is positively associated with teacher professional development.

#### 2.2.3 Teacher professional development and teacher performance.

Teachers’ participation in school-based training, teaching seminars, and other professional development activities helps improve teaching design, classroom implementation, and teaching reflection capabilities, thereby improving teaching effectiveness and student achievement [34]. Indicated that high-quality professional development is associated with stronger teaching skills, higher teacher self-efficacy and satisfaction, and better performance. [35]. Found that the frequency and quality of professional development were positively associated with teachers’ performance across multiple dimensions, such as research and teaching. [36]. Furthermore, prior research has shown that professional development is associated with teachers’ transformation of educational concepts and the development of reflective awareness, which are in turn linked to variations in teacher performance [37]. International research on teacher professional development highlights its contextual and relational nature. Large-scale reviews and empirical studies suggest that high-quality professional development is associated with instructional quality and teachers’ professional growth across diverse educational systems [38,39]. Comparative international evidence further underscores the importance of professional development as a sustained and collaborative process rather than isolated training activities [40]. Therefore, the findings of this study indicate a positive association between teacher professional development and teacher performance.

#### 2.2.4 Mediating role of teacher professional development between growth mindset and teacher performance.

Research indicates that teachers with a growth mindset tend to maintain more positive attitudes, which are positively associated with their participation in professional development activities [41,42]. Teacher professional development has been shown to be associated with teaching skills and is widely regarded as an important relational pathway linked to variations in teacher performance [43,44]. Existing empirical research confirms the connection between teacher psychological traits and professional development behaviors, offering valuable perspectives for understanding the relational pathways connecting these factors to teacher performance [45]. Therefore, the findings of this study suggest that growth mindset is indirectly associated with teacher performance through its association with teacher professional development, with professional development functioning as an associational mediator in this relationship.

### 2.3 Lifelong learning, teacher professional development, and teacher performance

#### 2.3.1 Lifelong learning and teacher performance.

Lifelong learning emphasizes individuals’ continuous updating of knowledge and skills throughout their careers to respond to educational environment changes [46]. Among teacher groups, lifelong learning is not only reflected in behaviors such as training, reflection, and professional reading but also represents an awareness of proactive growth and educational innovation [47]. Research indicates that teachers can effectively respond to teaching challenges and improve teaching quality and comprehensive performance through continuous learning [48]. Lifelong learning emphasizes teachers’ continuous updating of knowledge, skills, and concepts to adapt to educational environment changes. Teachers with lifelong learning tendencies are more dynamic and adaptable, with superior performance [49,50]. Therefore, this study suggests that teacher lifelong learning is positively associated with teacher performance.

#### 2.3.2 Teacher lifelong learning and teacher professional development.

Lifelong learning emphasizes individuals’ ability to continuously acquire and update knowledge throughout their careers [51]. Lifelong learning behaviors are manifested as teachers actively participating in curriculum training, teaching observation, academic seminars, and other activities, serving as key pathways for their professional development [52]. Teachers with lifelong learning awareness more actively participate in training, seminars, and reflection activities, continuously improving professional qualities and educational concepts, thereby promoting professional development [53]. Hayes et al. [54] survey shows that lifelong learning significantly correlates with teachers’ participation in high-quality professional development activities, helping improve classroom practice and career growth [54]. Furthermore, lifelong learning is not only reflected in continuous learning behaviors but is also a self-driven cognitive process that helps enhance teachers’ reflective abilities and professional identity [55,56]. Therefore, this study suggests that teacher lifelong learning is positively associated with teacher professional development.

#### 2.3.3 Mediating role of teacher professional development between lifelong learning and teacher performance.

Lifelong learning is an important concept for teachers to respond to educational reform and achieve continuous career growth, emphasizing continuous updating of knowledge and abilities throughout the entire career cycle [57]. Research indicates that teachers with stronger lifelong learning awareness tend to participate more actively in school-based training and teaching seminars, which are positively associated with their professional development [58,59]. Teacher professional development has been shown to be associated with teaching behaviors, teacher self-efficacy, and professional identity, and is widely regarded as an important relational pathway linked to variations in teacher performance [60,61]. Therefore, the findings of this study suggest that teacher professional development functions as an associational mediator linking teacher lifelong learning and teacher performance.

**Fig 1:**
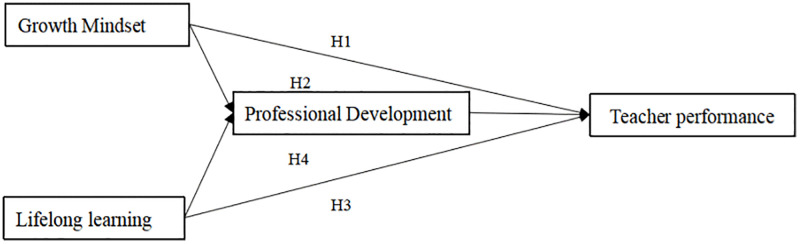
Research framework diagram.

Based on the above theoretical analysis and hypothesis inference, this study proposes the following hypotheses, as shown in Fig 1.

H1: Teacher growth mindset is positively associated with teacher performance.

H2: Teacher professional development serves as an associational mediator in the relationship between teacher growth mindset and teacher performance.

H3: Teacher lifelong learning is positively associated with teacher performance.

H4: Teacher professional development serves as an associational mediator in the relationship between teacher lifelong learning and teacher performance.

## 3. Method

### 3.1 Sample and data collection

This study employed a convenience sampling method to conduct an anonymous questionnaire survey among teachers from multiple universities. This study collected a total of 703 questionnaires. After removing invalid, missing, or irregularly answered questionnaires, 620 valid questionnaires were finally obtained, with an effective response rate of 88.2%. According to the structural equation model sample size calculation standard proposed by Cappelleri et al. [62], the sample size should be no less than 10 times the total number of questionnaire items. The questionnaire used in this study contains a total of 59 items, and the obtained effective sample size of 620 meets the minimum sample size requirement and is feasible for subsequent structural modeling analysis.

Among the 620 valid questionnaires, there were 215 male teachers (34.7%) and 405 female teachers (65.3%). Regarding age structure, 21–25 years accounted for 22.3%, 26–30 years 46.7%, 31–40 years 15.0%, 41–50 years 11.0%, 51–60 years 1.7%, and over 60 years 3.3%. In terms of education, bachelor’s degree 60.7%, master’s degree 28.0%, and doctoral degree 11.3%. Teaching experience of less than 3 years accounted for 22.3%, 4–10 years 47.0%, 11–20 years 18.0%, and over 21 years 12.7%. Regarding professional titles, teaching assistants 47.7%, lecturers 33.3%, associate professors 14.3%, and professors 4.7%. The sample covers different genders, ages, education levels, teaching experience, and professional title levels, demonstrating certain representativeness and diversity.

#### 3.1.1 Ethical approval and informed consent.

This study was reviewed and approved by the Ethics Committee of Hebei Hengshui University (Approval No. LLSC202512). All participants were adult volunteers and provided written informed consent prior to data collection. Participants were informed of the purpose of the study and the intended use of the data. All responses were anonymous, personal information was kept confidential, and the data were used solely for academic research. Data collection was conducted between 15 April 2025 and 10 June 2025.

### 3.2 Measures

#### 3.2.1 Teacher growth mindset scale.

The teacher growth mindset scale compiled by Sigmundsson and Haga was selected [63]. The scale was originally in English, and to ensure semantic accuracy and cultural adaptation of the Chinese translation, the study followed the “back-translation” method proposed by Brislin [64] for translation processing. The scale consists of 8 items in a single-dimensional structure. The scale uses a 5-point Likert scale (1 = strongly disagree; 5 = strongly agree), with higher scores indicating stronger teacher growth mindset. In this study, reliability analysis results showed that the scale’s Cronbach’s α value was 0.958, greater than 0.700, indicating good scale reliability [65]. Confirmatory factor analysis results showed GFI = 0.978, NFI = 0.983, IFI = 0.995, TLI = 0.993, CFI = 0.995, all reaching standards above 0.800, indicating good structural validity of the scale [66].

#### 3.2.2 Teacher lifelong learning scale.

The teacher lifelong learning scale compiled by Gür Erdoğan and Arsal was selected [67]. The scale was originally in English, and to ensure semantic accuracy and cultural adaptation of the Chinese translation, the study followed the “back-translation” method proposed by Brislin [64] for translation processing. The scale consists of 17 items in a two-dimensional structure, namely learning willingness and openness to improvement. The scale uses a 5-point Likert scale (1 = strongly disagree; 5 = strongly agree), with higher scores indicating stronger teacher lifelong learning ability. In this study, reliability analysis results showed that the scale’s Cronbach’s α value was 0.983, greater than 0.700, indicating good scale reliability [65]. Confirmatory factor analysis results showed GFI = 0.953, NFI = 0.973, IFI = 0.998, TLI = 0.998, CFI = 0.998, all reaching standards above 0.800, indicating good structural validity of the scale [66].

#### 3.2.3 Teacher professional development scale.

The teacher professional development scale compiled by Polatcan [68] was selected. The scale was originally in English, and to ensure semantic accuracy and cultural adaptation of the Chinese translation, the study followed the “back-translation” method proposed by Brislin [64] for translation processing. The scale consists of 17 items in a three-dimensional structure, namely keeping updated, reflective practice, and practice change. The scale uses a 5-point Likert scale (1 = strongly disagree; 5 = strongly agree), with higher scores indicating stronger teacher professional development. In this study, reliability analysis results showed that the scale’s Cronbach’s α value was 0.974, greater than 0.700, indicating good scale reliability [65]. Confirmatory factor analysis results showed GFI = 0.934, NFI = 0.943, IFI = 0.978, TLI = 0.974, CFI = 0.978, all reaching standards above 0.800, indicating good structural validity of the scale [66].

#### 3.2.4 Teacher performance scale.

The Chinese version of the teacher performance scale compiled by Yu et al. [69] was selected. The scale consists of 17 items in a three-dimensional structure, namely talent cultivation performance, research and academic performance, and social service performance. The scale uses a 5-point Likert scale (1 = strongly disagree; 5 = strongly agree), with higher scores indicating higher teacher performance. In this study, reliability analysis results showed that the scale’s Cronbach’s α value was 0.958, greater than 0.700, indicating good scale reliability [65]. Confirmatory factor analysis results showed GFI = 0.943, NFI = 0.964, IFI = 0.990, TLI = 0.998, CFI = 0.990, all reaching standards above 0.800, indicating good structural validity of the scale [66].

### 3.3 Statistical analysis

Data analysis was conducted using SPSS 22.0 and AMOS 21.0. First, reliability analysis and confirmatory factor analysis were used to test the reliability and validity of the measurement tools used in this study. Second, Harman’s One-Factor Test was used for common method bias testing. Third, descriptive analysis and Pearson correlation analysis were conducted to analyze participants’ overall performance on each variable and the degree of correlation between variables. Fourth, AMOS 21.0 was used for structural model testing.

## 4. Results

### 4.1 Common method bias

This study conducted common method bias testing using Harman’s single-factor test method, performing unrotated principal component factor analysis on all variable items. Four factors with eigenvalues greater than 1 were obtained, and the variance explained by the first factor was 43.751%, below the critical standard of 50%, indicating that common method bias problems in this study are not serious [70].

### 4.2 Correlations

This study conducted descriptive statistical analysis and correlation analysis on the four variables of teacher growth mindset, lifelong learning, professional development, and teacher performance. Results are shown in Table 1. Growth mindset and lifelong learning were significantly positively correlated (r = 0.215, p < 0.001); growth mindset and professional development were significantly positively correlated (r = 0.491, p < 0.001); growth mindset and teacher performance were significantly positively correlated (r = 0.289, p < 0.001); professional development and lifelong learning were significantly positively correlated (r = 0.568, p < 0.001); lifelong learning and teacher performance were significantly positively correlated (r = 0.257, p < 0.001); professional development and teacher performance were significantly positively correlated (r = 0.498, p < 0.001). The correlation coefficients between the four variables ranged from 0.215 to 0.568, indicating moderate to low correlations between variables with no serious collinearity problems [71].

**Table 1:** Pearson correlation.

	Growth Mindset	Lifelong Learning	Professional Development	Teacher Performance
Growth Mindset	1			
Lifelong Learning	0.215^***^	1		
Professional Development	0.491^***^	0.568^***^	1	
Teacher Performance	0.289^***^	0.257^***^	0.498^***^	1

Note:* p < 0.05 ** p < 0.01 *** p < 0.001.

### 4.3 Structural model

**Fig 2:**
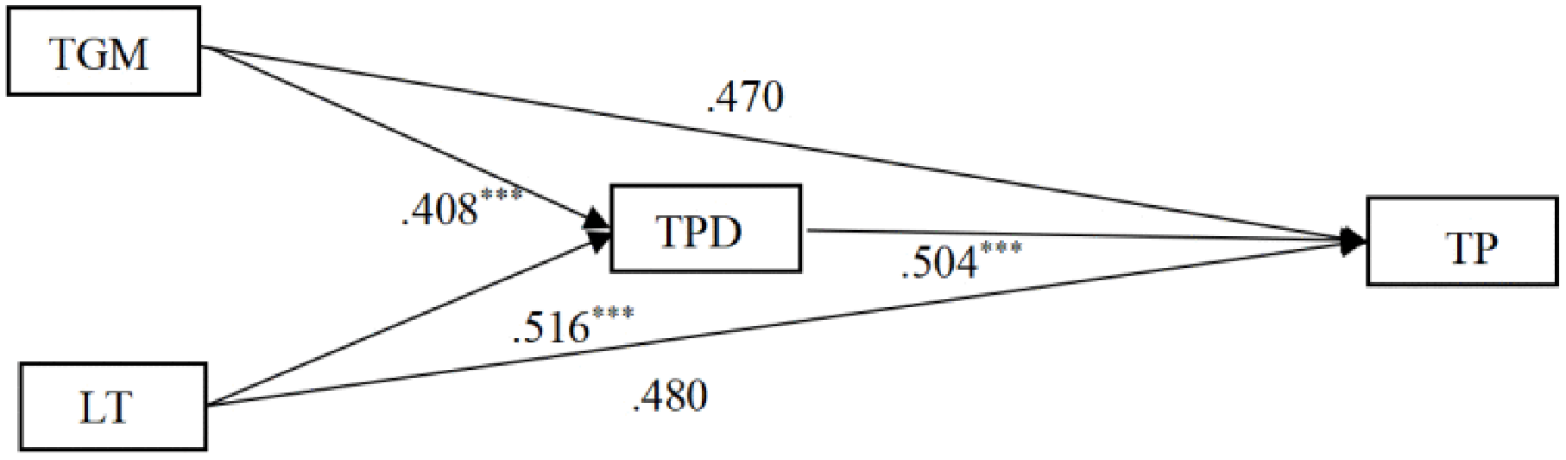
Structural equation model. p < .001*** TGM = Teacher Growth Mindset, LT = Lifelong Learning, TPD = Teacher Professional Development, TP = Teacher Performance.

As shown in Fig 2, this study examined the relationships between teacher mindset, lifelong learning, professional development, and teacher performance by constructing a structural equation model. Results showed χ²/df = 2.922, less than the standard of 5. Other fit indices were SRMR = 0.020, GFI = 0.977, AGFI = 0.954, PGFI = 0.642, NFI = 0.990, IFI = 0.993, TLI = 0.990, RFI = 0.984, CFI = 0.993. All fit indices reached acceptable levels [72]. The structural model explained approximately 46% of the variance in professional development (R² = 0.46) and 25% of the variance in teacher performance (R² = 0.25), indicating acceptable explanatory power [73]. All standardized factor loadings were statistically significant and exceeded recommended thresholds. The standardized loadings ranged from 0.87 to 0.89 for growth mindset, 0.88 to 0.91 for lifelong learning, 0.84 to 0.89 for professional development, and 0.77 to 0.84 for teacher performance, indicating adequate convergent validity [74]. In addition, The saturated model yielded an AIC value of 90.00 and a BIC value of 289.34, which were substantially lower than those of the independence model (AIC = 6689.13, BIC = 6729.00). These results indicate that the proposed model demonstrates good parsimony and fits the data considerably better than a null model [75].

To ensure robustness, this study further used the non-parametric percentile Bootstrap method for mediation path effect testing. Results showed that the 95% confidence intervals of bias-corrected non-parametric percentile direct effects, mediation effects, and total effects all did not include 0 (see Table 2). Results showed that teacher growth mindset was significantly and positively associated with teacher performance through its association with teacher professional development (β = 0.206, p < 0.001). Similarly, teacher lifelong learning was significantly and positively associated with teacher performance via teacher professional development (β = 0.260, p < 0.001).

**Table 2:** Indirect path effect summary.

Parameter	Effect	95% LLCI	95% ULCI
Indirect effect1	0.206^***^	0.149	0.284
Indirect effect2	0.260^***^	0.012	0.074
Total effect	0.257^***^	0.218	0.366

## 5. Discussion

This study found that neither teacher growth mindset nor lifelong learning showed significant direct associations with teacher performance, but both demonstrated significant indirect associations with teacher performance through their relationships with teacher professional development. Teacher professional development functioned as an associational mediator within the overall model, supporting related research by Lin et al. [20] and Heikkinen et al. [72]. This result indicates that teacher growth mindset and lifelong learning do not directly translate into teacher performance but their associations with performance appear to operate through their relationships with professional development behaviors in teaching practice. If teachers lack continuous professional development support, even with growth mindset beliefs and lifelong learning awareness, their intrinsic motivation is difficult to effectively translate into explicit performance improvement.

First, associational pathway linking teacher growth mindset and teacher performance through teacher professional development was verified. Tend to show higher levels of engagement actively seek professional development opportunities and proactively participate in teaching research activities and training programs, which are associated with stronger teaching competencies and reflection levels, this pathway is consistent with research by Lin et al. [20], Yeager and Dweck [10]. and can be interpreted through the lens of social constructivist learning theory: teachers continuously construct knowledge in social interaction and professional practice, translate internal beliefs into effective teaching-related behaviors [76].

Second, lifelong learning also improves performance levels through teacher professional development. Lifelong learners have tendencies for continuous learning and adaptation to updates, which enables them to demonstrate stronger initiative and efficacy in professional development processes, thereby optimizing teaching quality and research outcomes [12,13]. Consistent with this, Guskey [77] emphasized that teachers’ continuous development process is the core link connecting their learning motivation with teaching performance.

The above findings further highlight the bridging role of professional development in the associations between teacher cognitive and behavioral variables and teacher performance, suggest that variations in university teacher performance are not directly linked to individual motivational factors alone of individual motivation but are more closely associated with the opportunities and developmental support they receive for development in organizational environments. This not only deepens understanding of the relational processes underlying teacher performance but also provides practical references for universities in teacher training and support strategies: enhancing teacher performance appears to depend on how such initiative is translated into sustained teaching and research effectiveness through systematic professional development mechanisms.

This finding structurally confirms the complexity of teacher performance formation mechanisms, consistent with social constructivist learning theory viewpoints. Individual development cannot be separated from specific social practice and continuous reflection, highlighting the bridging role of teacher professional development between internal beliefs and external performance, providing more detailed pathway perspectives for understanding teacher growth mechanisms. Based on this, it is recommended that schools in teacher development work should not only emphasize the cultivation of growth mindset and lifelong learning attitudes but also construct systematic professional development mechanisms such as continuous training, teaching research collaboration, and professional communities to stimulate teachers to transform concepts into actual behaviors, thereby achieving sustainable performance improvement.

From a global perspective, this resonates strongly with international policy frameworks. For instance, UNESCO’s Teacher Development Framework and OECD’s Teaching and Learning International Survey (TALIS) both emphasize the importance of ongoing professional development as a key factor associated with teaching quality and student learning outcomes [12,78]. Our findings provide empirical support for these frameworks, showing how growth-oriented beliefs and lifelong learning tendencies can only be translated into measurable performance when supported by structured professional development mechanisms.

In the context of higher education worldwide, universities are increasingly held accountable not only for research output but also for teaching quality. The results of this study offer practical implications for global higher education institutions: cultivating growth mindset and lifelong learning orientations among faculty members must be coupled with systematic professional development programs—such as collaborative learning communities, mentoring systems, and cross-cultural training initiatives—to ensure sustainable performance improvement. This perspective transcends the Chinese context and offers valuable lessons for universities across both developed and developing countries striving to balance academic excellence with teaching effectiveness.

By situating teacher growth mindset and lifelong learning within a constructivist framework, this study further advances theoretical understanding of how cognitive beliefs and behavioral tendencies are socially enacted through professional communities and institutional mechanisms. It reinforces the notion that teacher performance is not an automatic result of motivation or belief, but rather the product of ongoing interaction between personal dispositions and systemic support structures. This provides valuable insights for educational policymakers, administrators, and practitioners seeking to strengthen higher education teaching quality on a global scale. These findings should be interpreted in light of several methodological limitations, which are discussed in the subsequent section.

## 6. Research contributions

### 6.1 Theoretical contributions

Based on social constructivist learning theory, this study reveals how teacher growth mindset and lifelong learning tendencies jointly affect teacher performance through professional development behaviors. This theoretical perspective emphasizes that teacher learning and development can be understood as dynamic processes shaped through social interaction, reflection, and practice, rather than solely as products of individual behaviors. Compared to previous research that examined growth mindset and lifelong learning separately as independent variables affecting teaching performance, this study incorporates both into a unified framework and introduces teacher professional development as a mediating variable, expanding the theoretical explanation pathways for teacher performance formation mechanisms. Meanwhile, this study focuses on university teacher groups, filling the empirical gap in teacher development mechanisms in higher education contexts based on existing basic education research, and providing new theoretical perspectives and structural modeling support for understanding the generation logic of university teacher performance.

### 6.2 Practical contributions

This study provides beneficial references for university education management practices, especially for formulating and implementing teacher performance improvement strategies. First, research indicates that teacher growth mindset and lifelong learning show significant indirect associations with performance, suggesting that universities should strengthen the cultivation and guidance of positive psychological traits in teacher team building. This can be achieved through special training, learning workshops, and growth-oriented leadership cultivation to guide teachers to establish beliefs that “abilities can be improved” and stimulate their intrinsic motivation for continuous learning and development. Second, research found that teacher professional development plays a core role in teacher psychological and behavioral pathways, indicating that relying solely on teachers’ personal willingness is insufficient to form synergy for performance improvement. Schools should construct more comprehensive teacher professional development support systems. For example, universities should further strengthen systematic support mechanisms for teacher professional development by providing personalized development planning, teaching ability improvement projects, and research guidance to enhance the targeting and effectiveness of teacher development. Furthermore, this study provides insights for optimizing university performance evaluation systems. Traditional performance evaluation over-focuses on outcome outputs and easily ignores teachers’ learning efforts and professional growth in teaching and research processes. Research results suggest that universities can appropriately incorporate indicators of teachers’ participation in professional development activities when formulating performance evaluation systems, guiding teachers to focus on process investment and enhancing the scientific nature and incentive effectiveness of performance evaluation.

Finally, this study offers practical insights for fostering a culture of lifelong learning among teachers. Universities may support continuous learning and reflective academic environments by developing learning organizations, establishing teacher learning communities, and encouraging cross-departmental teaching exchanges. Such initiatives are likely to be associated with more sustained professional development and may contribute to higher levels of teacher performance, thereby offering valuable reference points for universities seeking to enhance the quality of educational development.

To sum up: The practical implications drawn from this study are as follows:

(1) For Higher Education Institutions Worldwide: Universities should design professional development systems that go beyond ad-hoc training. Structured mentoring, collaborative learning communities, and cross-cultural teaching initiatives are necessary to ensure that teachers’ growth-oriented beliefs are translated into improved performance.
(2) For Policymakers and International Organizations: Our findings provide empirical support for UNESCO’s emphasis on lifelong teacher learning and OECD’s TALIS framework, both of which underscore the centrality of professional development. Policies should prioritize sustained investment in faculty learning opportunities, particularly in emerging contexts where institutional resources may be limited.
(3) For Teacher Educators and Practitioners: Teacher growth mindset and lifelong learning tendencies should be actively cultivated in professional development programs. Integrating reflective practice, peer coaching, and continuous feedback systems can help teachers leverage their dispositions in ways that are positively associated with performance.
(4) For Global Scholarship: While grounded in data from China, the study’s implications transcend national boundaries. By highlighting professional development as a key associational mediator, the research underscores the importance of cross-national dialogue and comparative studies to further validate these mechanisms in diverse higher education systems.

## 7. Conclusion

This study examined the relationships among teacher growth mindset, lifelong learning, professional development, and teacher performance in higher education. The results confirmed that growth mindset and lifelong learning are not directly associated with; rather, their associations with performance appear to operate through professional development. This finding advances our understanding of teacher performance by showing that individual beliefs and dispositions must be enacted through structured opportunities for continuous learning are linked to performance primarily when expressed through.

By situating the analysis within a constructivist learning framework, the study demonstrates that teacher performance is not simply the product of intrinsic motivation but is co-constructed through social, institutional, and cultural contexts. In doing so, the research contributes to global conversations on how higher education institutions can strategically support teachers in ways that align with international policy calls for sustainable and systemic professional development.

## 8. Limitations and future research directions

This study still has some limitations. First, this study targeted Chinese university teachers as research subjects. Although the sample has certain representativeness, the geographical and institutional types are relatively concentrated, mainly sourced from undergraduate institutions in some provinces. Therefore, the generalizability of research results may be limited. Future research can further expand sample composition to cover university teachers from different regions and different levels to enhance the universality and external validity of research conclusions. Second, this study used cross-sectional questionnaire survey methods, only presenting relationships between variables at specific time points, making it difficult to reveal the dynamic evolution of causal pathways. Although cross-sectional data has certain explanatory power in exploring relationships between variables [79], future research can still consider using longitudinal tracking designs to further verify the dynamic action mechanisms between growth mindset, lifelong learning, and teacher performance. Finally, this study focused on the mediation pathway of teacher growth mindset and lifelong learning associated with performance through teacher professional development, without incorporating possible moderating variables such as organizational support, teaching autonomy, and school cultural atmosphere. Future research can introduce these moderating factors into the model to explore the possible moderating roles of different contextual variables between teacher psychological traits and performance, to more comprehensively reveal the complex mechanisms of teacher performance formation.

This study relied on self-reported questionnaire data, which may be subject to social desirability bias and common method variance. Although validated instruments were used and Harman’s single-factor test suggested no serious common method bias, self-reported measures may not fully capture actual professional development engagement or performance-related behaviors. Future research could incorporate multi-source data, such as peer evaluations, administrative records, or observational measures, to enhance measurement robustness. Furthermore, the study was conducted within a specific cultural and institutional context. Cultural norms related to teacher learning, professional development practices, and performance evaluation may vary across countries and higher education systems, which may limit the generalizability of the findings. Replication studies in diverse cultural contexts and cross-cultural measurement invariance testing are recommended to assess the stability of the observed relationships.

## Supporting information

S1 File PLOS inclusivity in global research questionnaire. (PDF)

S2 File Minimal dataset underlying the findings (EXCEL). (XLSX)
